# Enhancing Volunteer Integration in Pediatric Care: Exploring Relationships, Facilitators, and Barriers

**DOI:** 10.5334/ijic.9042

**Published:** 2025-11-12

**Authors:** Federico De Luca, Silvia Mitidieri, Cristina Masella

**Affiliations:** 1 Department of Management Engineering, Department of Design, Politecnico di Milano, Milan, Italy; 2 Department of Management Engineering, Politecnico di Milano, Milan, Italy

**Keywords:** integrated care, volunteers, pediatric care, health care provision, family support

## Abstract

**Introduction:**

The integration of volunteers into healthcare has become increasingly relevant for improving patient care and addressing systemic resource constraints. In pediatric settings, volunteers offer essential emotional and personalized support. However, their collaboration with healthcare professionals is often hindered by challenges such as role ambiguity, limited space, and insufficient communication.

**Description:**

This study investigates the dynamics of collaboration between healthcare professionals and volunteers in pediatric hospital care. Drawing on narrative interviews with 25 volunteers from an Italian organization, it explores lived experiences and identifies key factors shaping volunteer-professional interactions. The findings are categorized into two main dimensions: *organizational arrangements* and *interpersonal dynamics*.

**Discussion:**

Facilitators of effective collaboration include temporal continuity, access to dedicated spaces, shared training initiatives, and improved communication. Barriers such as staff turnover, lack of formal recognition, and unclear role boundaries can undermine volunteer engagement. Informal relationship-building and structured information sharing were found to enhance cooperation and care quality.

**Conclusion:**

The study highlights the need to strengthen both structural and relational aspects of volunteer integration in pediatric care. By addressing these dynamics, healthcare institutions can enhance volunteer contributions, improve patient experience, and support the broader implementation of integrated care models.

## Introduction

The delivery of healthcare services plays a pivotal role in managing national services worldwide. The methods employed to provide these services differ, as do the allocations of resources [1]. However, a consistent focal point that has garnered increasing attention from policymakers is the active involvement of communities in various facets of the delivery system. While the forms and levels of participation may vary significantly, numerous government-sponsored healthcare delivery initiatives incorporating community engagement are being established and integrated into institutional frameworks [2].

This study adopts a social science-based definition of integrated care to highlight a comprehensive, multidimensional approach that extends beyond service delivery. By viewing integrated care as a coordinated framework of methods and models spanning funding, administration, organization, service delivery, and clinical levels, the study underscores the importance of fostering connectivity, alignment, and collaboration within and between care and cure sectors [3]. This approach aims to improve care quality, life quality, consumer satisfaction, and system efficiency by aligning services, providers, and settings across various levels, both within and beyond the healthcare sector [4].

In addressing the challenges posed by demographic shifts, staff shortages, and evolving societal needs in healthcare, there is a growing recognition of the necessity for innovative approaches. While volunteering has yet to be firmly established as a cornerstone of the welfare state in certain countries, some governments and nonprofit or community-based organizations advocate for increased incorporation of volunteers, leveraging the commitment of numerous dedicated individuals [5].

Volunteerism is central to managing healthcare organizations by offering the potential to integrate volunteers for paid staff in specific tasks [6,7]. Moreover, integrating volunteers into healthcare settings can improve the quality of care provided. While paid staff often face time constraints, volunteers typically possess the flexibility to dedicate more time to patients and their families. Research indicates that volunteers exhibit higher sociability and extroversion levels than their paid counterparts in similar roles [8,9].

Volunteers fulfill an essential function as coproducers in service delivery, providing effort, time, and information [10]. Effective collaboration between volunteers and professional staff is crucial for maintaining high service quality. Poor relationships between volunteers and staff can lead to decreased job satisfaction, increased stress, and higher turnover intentions among both groups. For volunteers, positive interpersonal relationships within the health organization foster continuous commitment. At the same time, dealing with external parties often causes tensions for paid staff, leading to negative consequences such as fear of reprisal and increased stress [11].

Models of volunteer resource management often emphasize motivation and relationships as the key drivers of efficiency and participation [12,13,14]. This is because issues related to tasks and responsibilities are typically considered more relevant for professionals and may risk compromising the spirit of volunteering.

Creating effective partnerships with voluntary organizations within care systems presents both operational and cultural challenges. These organizations often need help to balance governance participation with meeting public sector requirements. Aligning priorities is also tricky due to their varied commitments to specific groups [2]. While the literature extensively debates individual predispositions versus organizational constraints, there needs to be more understanding of how volunteer activities, initially driven by personal motivations, can be adapted to broader organizational frameworks. Further research is needed to determine how personal engagement can align with and strengthen organizational objectives in integrated care settings.

Karam et al. [15] and Schot et al. [16] highlight the hallmarks of effective integrated and inter-professional collaboration, which involve establishing suitable organizational arrangements and fostering an open and supportive professional culture. Key organizational arrangements include well-defined information structures, clear rules and responsibilities, and shared objectives. The professional culture is characterized by reciprocal trust, respect, mutual understanding, shared philosophies and values, and a readiness to collaborate and communicate effectively.

Nuño-Solinis et al. [17] propose a two-factor structure for evaluating collaboration, emphasizing interpersonal dynamics among professionals and the organizational environment’s characteristics. Palanisamy et al. [18] underscore the importance of interactional factors alongside organizational structures in facilitating effective collaboration. Furthermore, von Schnurbein et al. [19] emphasize the significance of appropriate organizational arrangements, such as a structured information system, clear rules and responsibilities, and alignment toward shared goals. They also stress the need for an open and receptive professional culture marked by reciprocal trust, mutual respect, familiarity among team members, and a willingness to collaborate and communicate.

Conflicts between paid staff and volunteers may arise from altruistic concerns about service quality or egoistic concerns regarding individual tasks or job security [19,20]. These conflicts can manifest as status conflicts, where volunteers assume tasks traditionally associated with professional roles, leading to perceived role compromise [20]. Process conflicts may occur due to role overlaps or volunteer non-compliance, potentially jeopardizing patient safety [19,20,21]. Task conflicts often emerge from differing opinions on task execution and prioritization, stemming from misunderstandings about each other’s roles and competencies [19,20]. Relationship conflicts may arise from personal incompatibilities or lack of recognition, often managed through avoidance strategies [9,19,20].

Understanding the dynamics of collaboration between volunteers and professionals offers valuable insights for shaping future policies regarding integrated and patient-centered care, enhancing patient care quality, and assessing the feasibility of integrating larger volunteer cohorts into professional healthcare settings [22].

This study aims to investigate the current collaboration between volunteers and professionals in hospital care, focusing on barriers and facilitators, and examines volunteers’ experiences assisting children and their families with severe illnesses in hospital care. The objectives are to elucidate: *What are the determinants of the dynamics of collaboration between volunteers and health professionals? What aspects should be considered for future implementation?*

## Methods

### Data collection

The study was conducted in collaboration with ABIO, a volunteer organization operating in Milan, Italy, which funded the project. This research is the second phase of a two-phase process, with the first phase contributing to inform the second. In the initial phase, interviews were conducted with families involved in the care process to gather their experiences and insights on services provided by the volunteer organization. These findings contributed to integrating the interviews for the second phase, which focused on volunteers. Data collection was rigorously controlled throughout the study, with all sensitive data anonymized, ensuring participant privacy and adherence to ethical standards. A qualitative research methodology based on narrative interviews was adopted to gather data [23]. Narrative interviewing prioritizes capturing the richness and context of participants’ lives through their narratives. This method contrasts starkly with quantitative approaches focusing solely on measuring specific variables. Researchers using this approach act as facilitators, creating a safe space where participants feel encouraged to share their stories. Non-verbal cues and attentive body language are fundamental in fostering openness. The interviewer practices active listening, allowing participants to lead the conversation and share their narratives without interruption, capturing the complexities of their lived experiences. After the narrative is shared, the interviewer shifts to an exploratory role, using open-ended questions framed in the participant’s language to probe deeper into details or clarify points. This approach encourages a collaborative dialogue, ensuring the participant’s voice stays central while giving the researcher a deeper understanding of their perspective. The open questions explored the volunteers’ journeys, motivations, initial and acquired skills, and the departments they had worked in. They examined relationships with colleagues and healthcare staff, highlighting synergies, challenges, and frustrations. They sought examples of role integration and suggestions for improving collaboration between volunteers and healthcare professionals to enhance the overall experience.

### Participants

The volunteers selected for this study belong to an organization with nearly 45 years of experience supporting children, adolescents, and families in hospitals within the Milan metropolitan area (Table 1). These volunteers undergo structured professional training, enabling them to offer emotional support, recreational activities, and a welcoming environment tailored specifically to pediatric patients and their caregivers. Additionally, the organization actively collaborates with healthcare facilities and local administrations to advocate for pediatric volunteerism and the rights of hospitalized children and their families.

**Table 1:** Information on the volunteers interviewed.


VOLUNTEER	EXPERIENCE *(IN YEARS)*	GENDER	HOSPITAL	AGE	DEPARTMENT	WORK

V1	1	F	A	50–60	Paediatric outpatient clinic	Yes

V2	1,5	F	B	50–60	Paediatric outpatient clinic	Yes

V3	1	M	C	40–50	Pediatric neuropsychiatry	Yes

V4	20	F	D	60>	Medium Intensity paediatrics	Retired

V5	1	F	E	60>	Paediatric outpatient clinic	Yes

V6	20	M	C	60>	Pediatric neuropsychiatry	Retired

V7	12	F	C	60>	Pediatric neuropsychiatry	Retired

V8	5	F	B	50–60	Emergency	Yes

V9	<1	F	B	60>	Paediatric outpatient clinic	Retired

V10	1	F	A	<40	Paediatric outpatient clinic	Yes

V11	4	F	F	60>	Paediatric surgery	Retired

V12	14	M	B	60>	Emergency	Retired

V13	1	F	A	40–50	Paediatric outpatient clinic	Yes

V14	5	M	B	50–60	Paediatric outpatient clinic	Yes

V15	1,5	F	F	<40	Paediatric surgery	Yes

V16	11	F	B	<40	Paediatric outpatient clinic	Yes

V17	1,5	F	F	50–60	Paediatric surgery	No

V18	5	F	D	50–60	Medium Intensity paediatrics	Yes

V19	1,5	M	C	60>	Pediatric neuropsychiatry	Retired

V20	7	F	E	50–60	Maternal and child pediatrics	Yes

V21	1	F	C	50–60	Pediatric neuropsychiatry	Retired

V22	6	F	B	<40	Emergency	Yes

V23	19	F	B	50–60	Emergency	Yes

V24	21	F	B	50–60	Emergency	Yes

V25	35	F	G	60>	Paediatric outpatient clinic	Retired


Although participants were not selected based on lived experience, several volunteers spontaneously shared personal or family-related motivations, enriching the findings with insights into the emotional and relational dimensions of pediatric volunteering. However, individuals with lived experience were not involved in the study’s design or implementation.

### Data analysis

The 25 interviews conducted were audio-recorded and transcribed verbatim. These transcripts represented the primary data source for the analysis. Coding was performed independently by two authors using NVivo software. To enhance methodological rigor and ensure inter-coder reliability, the codes generated by each author were compared. Discrepancies were discussed and resolved through iterative dialogue until consensus was reached, ensuring consistency in the identification of quotations and emerging themes.

Four key factors influencing volunteer–professional collaboration emerged, grouped into two macro-categories: *organizational arrangements* and *interpersonal dynamics* (Table 2).

**Table 2:** The 4 clusters emerged from the interviews.


ORGANIZATIONAL ARRANGEMENTS	INTERPERSONAL DYNAMICS

Temporal continuity	Relationships and integration

Spatial organization	Collaborative training


The identified themes within the “organizational arrangements” realm are temporal continuity and spatial organization. Temporal continuity refers to the seamless continuity of care between health professionals and volunteers, particularly regarding their shift changes. This continuity affects the patient’s perception of a coordinated care pathway and facilitates the transfer of information along their pathway [24]. Conversely, spatial organization pertains to the operational and physical context, mainly focusing on the spaces dedicated to volunteer activities. As Arnon et al. [25] highlighted, the physical elements of organizational resources, systems, and physical and technological spaces allocated to volunteer engagement can profoundly influence volunteers’ frustration, engagement, satisfaction, and retention over time [26,27].

Turning the attention to “interpersonal dynamics,” the themes that emerged from the interviews are relationships and integration, and collaborative training. Relationships and integration refer to the interactions between health professionals and volunteers, alongside the integration of volunteers into the healthcare environment [28]. The literature suggests that integrating volunteers into formal organizational environments can introduce various tensions despite its benefits, including conflicts between paid staff and volunteers and perceptions of role compromise [20,29,30], the challenge of balancing volunteer involvement with operational planning [31], reconciling qualifications with empathy [12], and managing the tension between authenticity and formalism [32]. However, the personal approach fostered by volunteer engagement creates a warmer and more welcoming atmosphere, ultimately increasing patient satisfaction [33]. Consequently, these improvements in the patient experience can confer a competitive advantage to the organization [34].

Finally, collaborative training encompasses the exchange of information between health professionals and volunteers during their joint training. The literature indicates that promoting information sharing, communication, and collaborative care planning between health professionals and volunteers is instrumental in facilitating care coordination [24,35], contributing to meeting the needs of service users and ensuring that they receive integrated, person-centered care across various settings, as highlighted by WHO [36].

Considering these four clusters, we categorized the nodes as barriers (B), facilitators (F), or suggestions (S) within each cluster (Table 3). Barriers and facilitators represent factors that either hinder or enhance collaboration between volunteers and healthcare staff, while suggestions encompass practical recommendations provided by volunteers to improve this collaboration. The coding was guided by the emotions and tones expressed during the interviews: frustration was linked to barriers, enthusiasm to facilitators, and aspirations to suggestions.

**Table 3:** Coded data from the volunteers interviewed.


VOLUNTEER	TEMPORAL CONTINUITY			SPATIAL ORGANIZATION			RELATIONSHIPS AND INTEGRATION			COLLABORATIVE TRAINING		

	B	F	S	B	F	S	B	F	S	B	F	S

V1	X	X	X				X	X	X			

V2		X					X	X	X			

V3							X	X	X			

V4	X		X				X		X			

V5	X			X			X	X	X		X	

V6							X	X				

V7							X	X	X			

V8	X				X		X		X	X	X	X

V9	X			X				X				

V10	X						X	X	X		X	X

V11									X			

V12				X			X	X	X			

V13	X						X	X			X	

V14			X	X			X	X	X			

V15			X		X		X		X			

V16		X					X		X			

V17			X		X		X	X			X	

V18	X				X		X					

V19		X			X			X			X	X

V20				X				X			X	

V21			X		X		X			X		

V22				X	X				X			

V23	X		X	X		X	X	X	X			X

V24	X			X	X		X		X	X	X	

V25					X		X		X		X	


## Results

Eleven recurring themes emerged from the analysis, corresponding to barriers, facilitators, and suggestions for volunteer-health professional collaboration, as listed in Table 4.

**Table 4:** Results classification.


ELEMENTS	FACILITATORS	BARRIERS	SUGGESTIONS

**Temporal continuity**		Work shift and continuity	Optimization of volunteer support based on scheduled hospital activities

**Spatial organization**		Lack of designated spaces	Organization of activities in suitable and protected spaces

**Relationships and integration**	Maintain discretion and respect privacy Foster informal engagement for a common goal Streamlining volunteer coordination with patient overview tools	Departmental dynamics and cooperation Concerns about disrupting healthcare staff, role overlap, and personal boundaries	Enhanced communication between clinicians and non-clinicians

**Collaborative training**		Challenges in volunteer training and capacity building	


### Facilitators

#### Maintain discretion and respect privacy

One of the most frequently mentioned concerns by the volunteers interviewed is the risk of breaching confidentiality, particularly through gossip, or that their presence in the hospital might inadvertently infringe upon the privacy rights of patients and healthcare professionals. This perception aligns with concerns documented in the literature, including Tavares et al. [21] and Karami et al. [30]. To mitigate such risks, volunteers are required to complete a structured and rigorous traineeship prior to formally taking on their roles, which serves to strengthen their awareness and professionalism in handling sensitive situations.

“You should not bother anyone; you should be discreet. Discretion was one of the qualities they asked us for at the beginning. With time, one can learn to be more discreet, understand how things work, become more familiar with the environment, and feel less like an outsider.” – V23.

#### Foster informal engagement for a common goal

The interviews highlighted a strong positive correlation between “Fostering a collective identity” and “Promoting trust and collaboration” with work engagement, significantly enhancing volunteer satisfaction. Volunteers expressed particular appreciation for instances where healthcare professionals informally collaborated for the child’s benefit, reflecting their alignment with these values. These findings are supported by Benevene et al. [37], whose study on Italian volunteers underscores the critical role of these dimensions in fostering effective volunteer engagement. Moreover, the research revealed that the link between these factors and volunteer satisfaction is fully mediated by work engagement, underlining important implications for volunteers’ organizational commitment [38].

“I have a great relationship with the healthcare staff. Some doctors have given me their cell phone numbers in case of urgent needs and have kindly helped me in various situations. In particular, I collaborated with a nurse, with whom I usually talk and joke, to administer eye drops to a child with neurological and mobility problems. We worked together because we wanted the best for the child.” – V2.

#### Streamlining volunteer coordination with patient overview tools

In some hospitals, compared to others, volunteers receive a list of hospitalized patients and a summary of their characteristics at the beginning of their shift. It helps to plan activities and provide support more effectively. According to volunteers working in hospitals where this practice is not adopted, such an approach is essential to avoid unpleasant misunderstandings.

“Even without delving into each child’s specifics, having a general overview like ‘this is a severe case, bedridden, while this one is mobile, and this one can eat independently, but that one requires assistance’ would be valuable. Our colleagues in [hospital name] compile a list of admitted children for the day. Simple details like ‘there are three children aged zero to three years, two boys and one girl, one speaks Italian, and the others do not’ could prove beneficial. This routine information provides a snapshot of the ward’s current situation upon arrival. This approach would let me determine whether a child would benefit from my presence in the playroom or if my attention should be directed elsewhere.” – V15.

### Barriers

#### Work shift and continuity

Ensuring continuity is crucial for providing a better experience for patients and their families while avoiding overlap and duplication in volunteers’ activities [28,35,42,43]. However, due to volunteers’ limited three-hour shifts spread across different time slots, there may be a need for coordination between volunteers and healthcare activities.

“Since we work the evening shift, we interact when the day ends. Additionally, the healthcare staff work in shifts, so when we go, we are not guaranteed to encounter the same nurses every time. Therefore, interacting with this type of staff is primarily an initial phase to understand if there are rooms where we absolutely cannot enter due to infectious children, which could pose a risk to our health, volunteers included.” – V4.

#### Lack of designated spaces

As Arnon et al. [24] and Taylor et al. [26] highlight, adequate physical space and facilities, including access to equipment, represent indicators of organizational investment, stability, clarity, and a sense of belonging. Volunteers who struggle with access to dedicated spaces may significantly reduce their volunteering hours, reporting both increased satisfaction when space is available and heightened dissatisfaction when it is lacking [39,40,41]. In our interviews, some volunteers report frustration over the lack or reduction of dedicated spaces, with some even choosing to leave due to the inadequate conditions. Over the years, frequent changes to the space layout and furnishings have resulted in reduced space, fewer lockers, and downsized tables. Despite renovations, space remains tight, leading to challenges in maintaining essential belongings and a sense of permanence in their work environment.

“We’re practically ‘camped out’ in the waiting room. Over the past twenty years, furnishings have changed multiple times, often putting our belongings at risk. Even after the latest renovation, space has shrunk, more chairs were added, but we lost a locker and the table was downsized.” – V24.

“The same room has been rearranged with new furnishings, but space is limited. Fortunately, the vending machines, long a source of complaints, were finally removed.” – V23.

The pandemic health emergency prompted organizations to reassess their offerings and internal structure. However, support activities not directly related to the clinical field, such as volunteering, have been sidelined during the post-pandemic recovery. As a result, some volunteers have expressed frustration in reclaiming appropriate spaces for recreational and playful activities with children now that the emergency has ended.

“After the pandemic, our dedicated playroom was repurposed into office space for medical staff, requiring us to use a lunchroom temporarily adapted for recreational activities. This change significantly reduced our sense of having an exclusive and functional space for volunteering.” – V14.

#### Departmental dynamics and cooperation

Significant variations in operational organization, timing, and space were observed in the hospital units examined in the study. This diversification affects relationships with staff in specific contexts, isolating the volunteer in their contribution. As emphasized, collaboration is highly fragile in emergencies or when patient stays are reduced.

“Our interactions with the nursing and medical staff are minimal in the emergency room, as we mostly wait in the reception area, which is usually restricted. Compared to other departments, there’s a clear lack of collaboration and communication. We used to ask questions at the start of our shifts, but the interaction usually ended there.” – V8.

#### Concerns about disrupting healthcare staff, role overlap, and personal boundaries

Volunteer activities are often disconnected from those of healthcare professionals, whose tasks may not align with volunteer demands. The discretionary nature of working with volunteers and the lack of explicit role definitions lead to a partnership primarily driven by individual willingness and current workload during interactions. The absence of a clear understanding regarding the roles and responsibilities of volunteers, including their limitations, fosters competition and apprehension among healthcare staff. As outlined in the literature review conducted by Studer and von Schnurbein [12], the behavior of healthcare professionals toward volunteers and the management of volunteer activities are guided by both explicit and implicit understandings of these dynamics. When actual behaviors deviate from these understandings, conflicts and disagreements can arise. These conflicts may originate from various factors, including competition for roles, increased workloads for paid staff resulting from volunteer involvement, concerns regarding service quality, inadequate acknowledgment of volunteers’ contributions, communication deficiencies, trust issues, ambiguous goal setting, divergent prioritization of objectives, and disputes over organizational identity.

“I do not know if they fear we will invade their territory or ask for collaboration, so there is always some tension. We do not want to feel like we are competing or in rivalry. I am also considering what we, as volunteers, could offer, but it is difficult because of legal constraints.” – V2.

“Some nurses did not welcome our presence, particularly during dinner when we entered the rooms, and children could become unsettled. In some instances, nurses issued an ultimatum, which was undoubtedly frustrating because we were not seeking to replace their role or engage in therapeutic activities but rather to provide emotional support, such as reading a storybook.” – V4.

Vanderstichelen et al. [22] also noted that limited access to nurses creates a barrier for volunteers, restricting their ability to provide effective support and coordinate care across various settings. Access is needed to ensure information sharing, risking the omission of critical details during shift changes.

“When a patient requests the presence of a volunteer, the hospital staff contacts us with a ‘Madam, you can go there.’ They call us even when we need to stay with the child because the mother needs to discuss with them. For the rest, we manage on our own. However, when I asked the head nurse if there was any news, and she responded negatively, she took no action. At most, she informs us that there is a COVID-19 patient in a room and asks us to keep our distance. I prefer more collaboration with them.” – V25.

#### Challenges in volunteer training and capacity building

In all the settings considered in this study, volunteers are only occasionally involved in light training sessions on common pathologies, symptoms, and procedures to follow in case of unexpected events. These initiatives are not held regularly and often lack continuity. Moreover, the training content is frequently not adapted for a non-medical audience, making it overly technical and difficult for volunteers to apply in practice. Although volunteers consistently express interest in receiving such training, its scheduling varies significantly across settings and is often disrupted by organizational changes, particularly shifts in healthcare leadership.

“As I mentioned, these initiatives often get interrupted due to changes in healthcare management, with changes in the head nurse or the chief physician, which can compromise the work already established.” – V8.

“This year, upon our request, we were informed about an advanced seminar that we could have attended, either in person or online, if we had wanted to. It was very technical, so drawing concrete conclusions was difficult.” – V21.

### Suggestions

#### Optimization of volunteer support based on scheduled hospital activities

Volunteer activities are coordinated informally, with volunteers adapting to the hospital’s routines. However, they recognize the value of closer collaboration and actively adjust their tasks to align more effectively with healthcare professionals to improve the overall patient experience.

“Sometimes, I struggled on Saturdays at noon due to delays caused by the shift change of the medical staff, which resulted in delays in visits. I remember leaving around one PM while many patients were still waiting, and it was difficult to explain the shift change to them. I would have liked to know more about the emergency room’s functioning and have greater staff involvement. Another idea would be to have volunteers assist children during triage to calm them without interfering with the work of doctors and nurses. Greater interaction would be useful and interesting.” – V23.

Operational efficiency can be enhanced by providing volunteers with a list of hospitalized patients and a summary of their characteristics at the beginning of each shift. Although only sometimes adopted, this practice is essential for effective planning and support. Volunteers report that such information helps avoid misunderstandings and ensures that their activities are tailored to the specific needs of the children, thereby optimizing their contributions.

#### Organization of activities in suitable and protected spaces

The allocation of space for volunteer activities should create a comforting environment for the child while integrating seamlessly with the surrounding setting to support those passing through. In high-turnover areas like the emergency room, volunteers recommend designing spaces tailored to this context’s specific needs and demands, which differ significantly from those in a pediatric ward or outpatient clinic.

“Volunteers in the emergency room: It would be beneficial if the space had been designed to be more welcoming and functional, even for those who have to spend time there, like us, who remain there for about 2/3 hours. However, the space is so limited that it is difficult not to immediately understand that there is a triage and a waiting room. More information or details upon arrival would be helpful, especially for those who are in that context for the first time and may feel lost.” – V23.

#### Enhanced communication between clinicians and non-clinicians

Vanderstichelen et al. [22] underscore the importance that volunteers place on regular, structured meetings involving coordinators, nurses, and other healthcare professionals to facilitate task management and conflict resolution. Interviews reveal a desire among volunteers for greater inclusion in such meetings, highlighting the potential benefits of a more collaborative approach.

“A meeting between volunteers and the healthcare staff could be a good idea. The respective heads meet, but it seems more like a top-down approach. I have never participated in such a meeting, nor have I ever inquired about it. I wonder if there is a meeting that involves everyone. Organizing a departmental event might be challenging, but bringing the two groups closer could be beneficial through some form of informal gathering.” – V2.

Emergencies can cause considerable stress to volunteers. Receiving training on frequent pathologies and their related symptoms allows them to manage such stressful situations better.

“I had also asked my supervisor if it was possible to have training sessions with the doctors, and we did it once. They also explained the particularities of some diseases, so we were prepared. I remember once at the [hospital name], there was a mother who had a child admitted in June. It is a somewhat particular case. Moreover, I was on duty; she had to leave to go to the bathroom. Furthermore, the child had a little apnea crisis, so the machines immediately started beeping. I have to tell the truth that I was scared, but as soon as the machine beeped, the nurse and the medical staff arrived immediately, so there was no problem.” – V19.

## Discussion

Based on the results of our interviews, we identified the primary challenges for effectively integrating volunteers into healthcare settings. These include the need for stable coordination mechanisms to ensure continuity of care amidst frequent staff turnover and the critical importance of dedicated, functional spaces that enhance volunteer efficacy. Building trust and fostering collaboration between volunteers and healthcare staff is essential for enhancing care quality. Furthermore, accessible, well-designed training programs are vital to equip volunteers with the competencies required for practical support in clinical environments. Each section provides strategies to strengthen volunteer roles and value their contributions within healthcare systems.

### Temporal Continuity

Ensuring task continuity is crucial for providing optimal patient and family experiences [42,43] while avoiding overlaps in volunteers’ activities [28]. Our research participants reported that their limited turnover across different time slots necessitates effective coordination with healthcare staff, who may also experience high turnover, as Eriksson et al. [44] highlight. This inconsistency can lead to communication gaps, disrupting care flow and impacting volunteer engagement and patient satisfaction, as Hudson [45] pointed out.

Staff turnover complicates trust relationships between volunteers and departmental managers [46]. In our study, this factor emerged as critical, mainly due to frequent changes in organizational leadership, which can interrupt established rapport, as new leaders may not be familiar with or invested in volunteers’ roles, as highlighted by Øygarden et al. [47]. This lack of continuity can diminish volunteer engagement and effectiveness, as stable and supportive relationships with staff are crucial for volunteers to feel valued and integrated [25,45].

Priority-based scheduling further limits volunteer access to healthcare staff, hindering support, care coordination, and information sharing during shift changes, increasing the risk of communication breakdowns. Consequently, results show that volunteers often manage independently with minimal updates, reducing their effectiveness. Crookes et al. [48] emphasize that developing organizational mechanisms to support information exchange is essential for effective communication between health professionals and volunteers.

For family caregivers and patients, a seamless experience is facilitated by the effective coordination of information between volunteers and healthcare personnel. Patients and their families, often in unfamiliar and stressful situations, benefit significantly from continuous emotional support and care provided by well-informed volunteers.

### Spatial Organization

Our interviewees suggest that creating comfortable spaces for children and seamlessly integrating them with the surrounding environment can facilitate navigation for all visitors. This is especially important in high-turnover areas like emergency rooms, where needs differ from those in pediatric wards or outpatient clinics. Volunteers also emphasized the need for functional and welcoming areas. Limitations, such as cramped spaces and lack of clear information upon arrival, can cause confusion and discomfort, particularly for first-time visitors. These suggestions are supported by Arnon et al. [25] and Taylor et al. [27], who state that allocating space for volunteer activities is critical for enhancing volunteer partnerships in healthcare.

Furthermore, healthcare organizations often need to recognize the importance of dedicated spaces for volunteer activities, exacerbated by the reorganization necessitated by the COVID-19 pandemic [49].

As reported by Purwanto & Rostiani [50] and Retzer [51], volunteers reported that during the post-pandemic recovery, some support activities had been deprioritized, leading to frustration among volunteers struggling to regain suitable spaces for their activities.

The lack of designated spaces impacts the efficiency and satisfaction of volunteers and reflects organizational investment and stability [40].

These issues underline the need for healthcare organizations to recognize and address the spatial requirements of volunteer activities. By ensuring that volunteers have access to well-defined, functional, and welcoming spaces, healthcare facilities can enhance the overall effectiveness and satisfaction of volunteer services, ultimately improving the quality of patient care. This approach requires reevaluating space allocation policies and a commitment to integrating volunteer needs into the broader organizational infrastructure.

### Relationships and Integration

In our study, volunteers emphasized the need for recognition from department heads to promote effective collaboration. Introducing new and existing volunteers to healthcare staff helps build trust and ensures volunteers are seen as reliable contributors to patient care, as van Bochove et al. [33] also stress.

The possibility for collaboration often hinges on the personal willingness of staff members and the workload during interactions [34]. Our research shows that volunteers experience varying levels of engagement, with some staff members exchanging chats and updates, while others maintain a more distant relationship. This dynamic highlights the importance of personal affinity in fostering meaningful interactions, regardless of the setting. Volunteers who feel a stronger personal connection with healthcare staff are more likely to feel integrated and effective in their roles [21].

This study revealed significant differences in operational organization, timing, and space allocation, which affect relationships with healthcare staff. In some departments, especially in emergency settings, volunteers report minimal interaction with staff, as their activities are confined to reception areas. This lack of effective partnerships hampers collaboration and limits the volunteers’ ability to contribute effectively. As discussed earlier, staff turnover is another challenge that affects the continuity of trust relationships between volunteers and departmental managers.

Our results show that concerns about overstepping their role or bothering healthcare staff stem from a lack of a clear understanding of volunteer roles and responsibilities, as emphasized by Studer & von Schnurbein [12], Koivula & Karttunen [34], and van Bochove et al. [33]. Interviewees suggest that effective management of volunteer activities, guided by clear and explicit understandings, is necessary to mitigate conflicts and enhance cooperation. Informal collaboration and a positive community atmosphere significantly contribute to volunteers’ work engagement and facilitate information sharing. Regular meetings involving coordinators, nurses, and other professionals are crucial for managing tasks and addressing conflicts. These meetings provide a platform for discussing challenges, sharing successes, and planning future activities.

### Collaborative Training

Respondents consider the involvement of volunteers in training sessions on common pathologies, symptoms, and emergency procedures crucial. However, it is infrequent and often not adapted for non-medical audiences, making it overly technical and less accessible. Despite volunteers frequently requesting such training, inconsistent scheduling influenced by contextual and internal policy changes poses a significant challenge. Our interviews reveal high expectations for training and indicate that private hospitals generally allocate more resources and attention to organizing these sessions.

Enhanced training can significantly increase volunteer awareness and their ability to manage stress during emergencies [52,53]. Ensuring volunteers are better prepared to support clinical teams during emergencies and handle critical situations more effectively underscores the need for collaborative, structured programs tailored to volunteers’ needs. This is essential for improving healthcare service efficacy.

## Conclusions

This study provides valuable insights into volunteer integration within pediatric healthcare settings. It highlights the barriers and facilitators that influence effective collaboration and offers practical suggestions based on volunteers’ experiences. The findings contribute to a deeper understanding of volunteers’ expectations and dynamics in hospital care while identifying critical aspects for future research.

Two primary obstacles in the collaboration between volunteers and professionals have been identified: the need for tools to facilitate patient information sharing and the implementation of effective leadership behaviors, as defined by Yukl et al. [54].

From a process perspective, detailed patient information, such as at the beginning of each shift and regular meetings between volunteers and healthcare staff, is important to improve operational efficiency. These practices are crucial for effective task management and conflict resolution. Implementing measures and tools that facilitate better communication, coordination, and integration of volunteer services will ultimately benefit patients, families, and healthcare teams.

Regarding leadership behaviors, volunteers and healthcare professionals represent distinct resources within the organization, leading to varying perceptions of their relationship. Organizations can foster an environment conducive to volunteer engagement by understanding and addressing these disparities. It is essential to consider these contextual factors when expanding volunteer roles and anticipating their interactions with healthcare professionals.

Healthcare organizations should collaborate with volunteer management staff to design targeted training programs that equip individual volunteers with the skills and knowledge needed to perform their roles within the hospital setting effectively. The training should be accessible and relevant to non-medical audiences, enhancing volunteers’ ability to manage stress and handle critical situations effectively.

In conclusion, addressing the organizational and interpersonal dynamics influencing volunteer integration is essential for optimizing their contributions and enhancing patient care. By fostering trust, providing adequate training and support, and promoting better communication, healthcare organizations can create a more supportive and effective environment for volunteers and healthcare professionals, improving patient outcomes and overall satisfaction.

A significant limitation of this study is that the interviews were exclusively conducted with members of a single volunteer organization and were confined to pediatric departments. This narrow scope may affect the representativeness of the findings and limit the ability to generalize the conclusions.

Most volunteers (19/25) were aged over 50, bringing valuable interpersonal maturity, emotional intelligence, and resilience, which are particularly beneficial for empathetic, patient-centered support in pediatric care. Their extensive lived experiences help them effectively navigate challenging emotional interactions with children and families in distress. Future research could explore structured initiatives, such as intergenerational mentoring or reflective storytelling, to explicitly leverage and share this accumulated wisdom, further enriching volunteer integration within healthcare organizations.

## Additional File

The additional file for this article can be found as follows:

10.5334/ijic.9042.s1 Supplementary File. Narrative Interview Methods.
