# Supplementary material for: Enhancing Volunteer Integration in Pediatric Care: Exploring Relationships, Facilitators, and Barriers
Source: Int J Integr Care. 2025 Nov 12;25(4):12. doi: 10.5334/ijic.9042 (PMC12617419; doi:10.5334/ijic.9042)
Supplement: Supplementary File. — Narrative Interview Methods. [file ijic-25-4-9042-s1.pdf]

Narrative Interview Methods

Participants: [Hidden Organization Name] volunteers who expressed availability during the meeting on 2/12/2023

Objective: Collection of experiences concerning cases of formal and informal integration/non-integration among [Hidden Organization Name] volunteers

Methodology and Practical Impact:

Narrative interviews (Anderson & Kirkpatrick, 2016) represent a method to gain deeper understanding of individuals' experiences and behaviors in relation to health contexts and illness, through their personal stories. Although this approach is primarily aimed at patients, it is noteworthy that volunteers are also influenced by the environment in which they operate. Literature suggests that the main motivation of volunteers is either their direct experience with illness, typical of patient-volunteers, or the presence of family members affected by conditions requiring care (Yeung et al., 2018; Pilmer et al., 1996).

The interview is structured in four sections:

1. **Introduction**
   During the introduction, the interview process, objectives, and data handling conditions for research purposes are explained.
2. **Narration**
   The participant begins by telling their story; the interviewer should encourage spontaneous conversation through nonverbal cues such as smiles and expressions of interest. The interviewer avoids interrupting until clear signals of story completion emerge.
3. **Narrative/semi-structured interview phase**
   At this stage, active listening by the interviewer is essential. The participant's language may be used to delve deeper into specific topics or to request further details. Questions should primarily aim at eliciting additional information, for example: "What happened next?" or "Can you elaborate on…?", avoiding questions about opinions or motives. Below is a possible thematic map of the interview as proposed by Gonella et al. (2019):

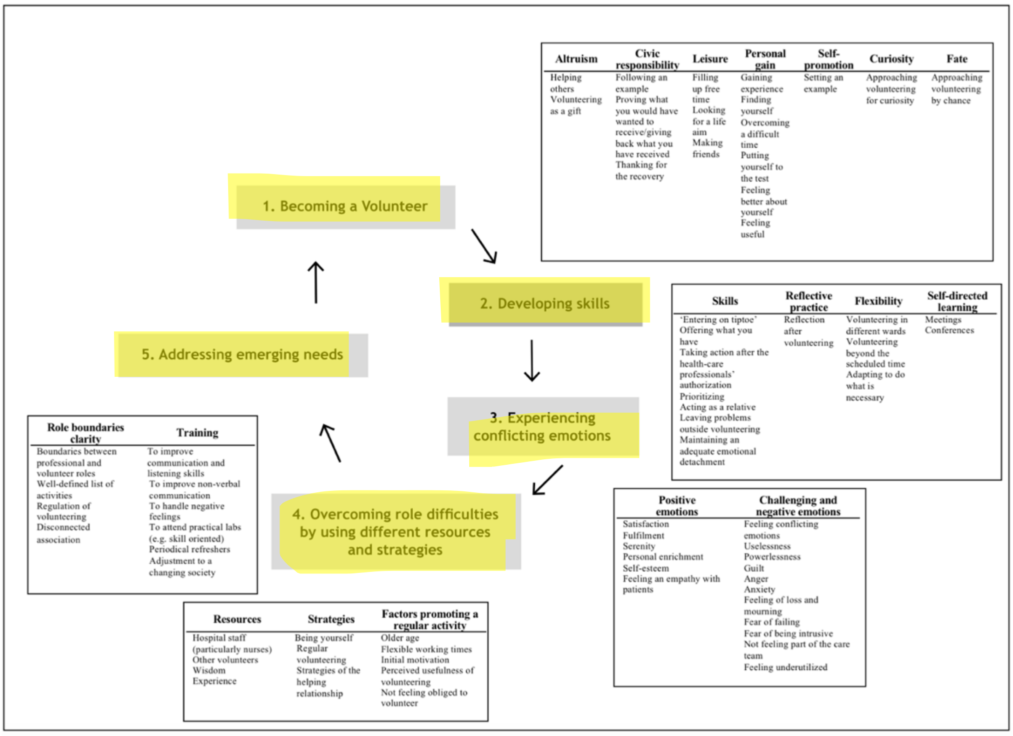

Figure 1. Themes, categories and codes as emerged from narratives.

4. **Conclusion**

   Finally, the interviewer concludes the interview and explains the next steps, such as the transcription process or the possibility for further contributions from the participant.

Estimated duration: 30–40 minutes

References:

Anderson, C., & Kirkpatrick, S. (2016). Narrative interviewing. *International Journal of Clinical Pharmacy, 38*, 631–634. https://doi.org/10.1007/s11096-015-0222-0

Gonella, S., Canzan, F., Larghero, E., Ambrosi, E., Palese, A., & Dimonte, V. (2019). Hospital Volunteering Experiences Suggest that New Policies are Needed to Promote their Integration in Daily Care: Findings from a Qualitative Study. *Zdr Varst, 58(4)*, 164-172. https://doi.org/10.2478/sjph-2019-0021

Pillemer, K., Landreneau, L. T., & Suitor, J. J. (1996). Volunteers in a peer support project for caregivers: What motivates them? *American Journal of Alzheimer's Disease, 11(*5), 13-19. https://doi.org/10.1177/153331759601100504

Yeung, K., Zhang, J. W., Zhang, Z., & Kim, T. Y. (2018). Volunteering and health benefits in general adults: Cumulative effects and forms. *BMC Public Health, 18*. https://doi.org/10.1186/s12889-017-4561-8

Narrative Interviews with Volunteers

Estimated duration: 40 minutes

Icebreaker (2 min)

Good morning, how are you?

Interviewer introduction and purpose of the interview (2 min)

[Hidden information]. I work on innovation in healthcare services through co-production and co-design. Currently, my research focuses on the dynamics of service co-production between volunteers (representing the informal sector) and healthcare and social care personnel (representing the formal sector). Through your experience, today's interview aims to improve understanding of the dynamics, the integration that has occurred or been lacking, and expectations and motivations for the future.

Narrative question outline (40 min)

1. How long have you been a volunteer, and what motivated you to become one?
   What steps did you follow to become a volunteer?
2. What skills did you have at the beginning, and which have you acquired over time?
3. In which departments have you performed volunteer activities?
4. What challenges or situations do you find most frustrating?
   Could you share some experiences?
5. How do you relate to your fellow volunteers?
6. What is your relationship with healthcare personnel?
   With which staff member do you have the greatest synergy? And with whom less?
7. Can you describe situations where your role integrated with that of the healthcare personnel?
8. What are the main challenges in collaboration between volunteers and healthcare personnel?
9. What improvements would you suggest to optimize this volunteer experience?
